# Spatial epidemiological characteristics and exponential smoothing model application of tuberculosis in Qinghai Plateau, China

**DOI:** 10.1017/S0950268822000036

**Published:** 2022-01-12

**Authors:** Y. Shang, T. T. Zhang, Z. F. Wang, B. Z. Ma, N. Yang, Y. T. Qiu, B. Li, Q. Zhang, Q. L. Huang, K. Y. Liu

**Affiliations:** 1 Department of Public Health, Qinghai University, Xining, Qinghai 810001, China; 2 Qinghai Center for Disease Prevention and Control, Xining, Qinghai 810007, China

**Keywords:** Cases clusters, exponential smoothing model, Qinghai Plateau, spatial − temporal distribution, tuberculosis

## Abstract

The epidemic of tuberculosis has posed a serious burden in Qinghai province, it is necessary to clarify the epidemiological characteristics and spatial-temporal distribution of TB for future prevention and control measures. We used descriptive epidemiological methods and spatial statistical analysis including spatial correlation and spatial-temporal analysis in this study. Furthermore, we applied an exponential smoothing model for TB epidemiological trend forecasting. Of 43 859 TB cases, the sex ratio was 1.27:1 (M:F), and the average annual TB registered incidence was 70.00/100 000 of 2009–2019. More cases were reported in March and April, and the worst TB stricken regions were the prefectures of Golog and Yushu. High TB registered incidences were seen in males, farmers and herdsmen, Tibetans, or elderly people. 7132 cases were intractable, which were recurrent, drug resistant, or co-infected with other infections. Three likely cases clusters with significant high risk were found by spatial-temporal scan on data of 2009–2019. The exponential smoothing winters' additive model was selected as the best-fitting model to forecast monthly TB cases in the future. This research indicated that TB in Qinghai is still a serious threaten to the local residents' health. Multi-departmental collaboration and funds special for TB treatments and control are still needed, and the exponential smoothing model is promising which could be applied for forecasting of TB epidemic trend in this high-altitude province.

## Introduction

Tuberculosis (TB) is a chronic infectious disease which results in the most human deaths from a single source of infection, and approximately 9.96 million new TB patients were reported worldwide in 2019 [1]. In addition, mixed infection of TB and HIV, drug resistance or multi-drug resistance, has brought huge challenges to TB's control [2–4]. It was reported that 6.5 billion dollars were used for prevention and treatment and 0.91 billion dollar for scientific research on TB all over the world in 2019. TB is a major public health problem with the highest absolute number of cases worldwide. China is one of the 30 countries with a high burden of tuberculosis, especially in western China [5–7]. Qinghai province locates in the northeast of the Qinghai-Tibet Plateau including two cities and six Tibetan autonomous prefectures, where more than 90% of the regions are mountains and grasslands with about half of the population living in this rural area. This plateau has an average altitude of over 3000 meters, which results in low oxygen, low pressure, cold and dry climate. A harsh natural environment and inconvenient traffic and relatively inadequate health resources lead to a high incidence of TB and more diagnostic delays of TB cases [8, 9].

For achieving the World Health Organization's goal of eliminating TB, Qinghai provincial government issued a series of programmers and plans for TB prevention and control [10, 11], demanding health-related departments and health workers to strengthen TB patients management, screen early TB cases, test drug resistance, optimize treatment protocols, develop case finding techniques. Several studies about the application of describing epidemiological methods had been reported, however, most of them only showed certain characteristics without a comprehensive consideration of the spatial-temporal information of diseases. Given this, we collected data of TB cases from 2009 to 2019 in Qinghai Plateau and analyzed epidemiological characteristics and spatial-temporal distribution of TB. The spatial-temporal analysis could accurately display TB case aggregation information visually and intuitively in the region, time, and number, so high-suffered areas of the TB epidemic could be determined. Based on this, the government would strengthen monitoring, prevention and control in these high-risk areas and populations. Furthermore, we built an exponential smoothing model for TB epidemiological trend forecasting, the interval of the epidemic intensity index would be foreseen, and then health institutions could reserve sufficient medical resources and personnel so as to achieve effective control.

## Methods

### Data sources

**Fig. 1.**
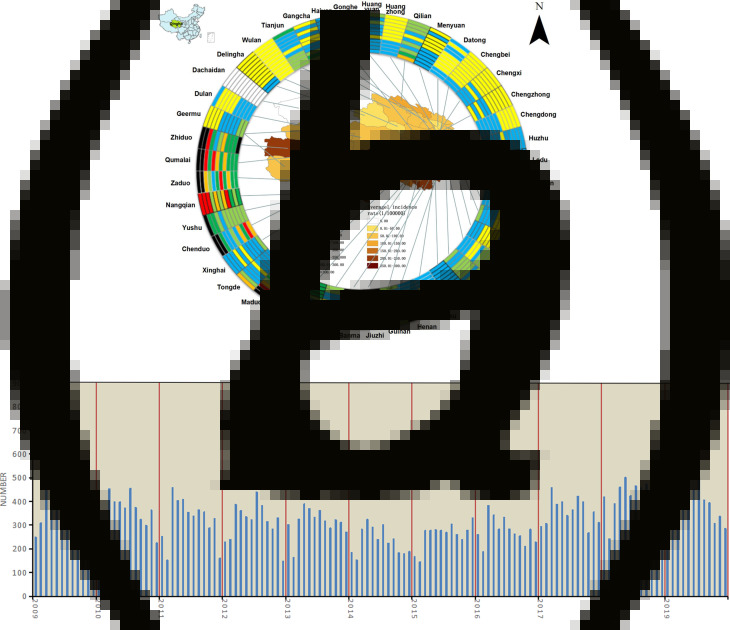
(a) The spatial distribution of the TB registered incidence (per 100 000 population) of 44 counties from 2009 to 2019 in Qinghai Province. The map inside the ring indicated the annual average registered incidence rate in the county-level. External rings indicated annual registered incidences in each specific county year by year (2009–2019) from the inside to the outside. Different incidence rates were marked with different colors, as shown in the legends. All counties were shown and linked to their corresponding locations on a map with lines. Dachaidan has no confirmed case, and the average annual registered incidence rate varied 26.77 per 100 000 to 270.80 per 100 000, annual registered incidence rate varied 5.31 per 100 000 to 590.03 per 100 000. (b) The registered monthly TB cases from 2009 to 2019 in Qinghai Province. The number varied from 128 to 848.

The registered tuberculosis cases data of Qinghai from 2009 to 2019 in regarding to regions (cities, districts or counties), sex, age, occupation, date of diagnosis, ethnicity and others were provided by Qinghai Center for Disease Prevention and Control (Qinghai CDC) from the TB Information Management System (TBIMS), which is an additional web-based national TB surveillance system developed by China, started at 1 January 2005, to which all TB health institutions are required to report diagnosed TB cases [12]. TB cases The demographic data from 2009 to 2019 that supported the analysis of this study were collected in Qinghai Provincial Statistical Yearbook [13]. This study covered two cities and six autonomous prefectures including 44 counties or districts (Fig. 1a), and had been reviewed by the Human Experiment Ethics Committee of Qinghai University School of Medicine (2018–09).

### Statistical analysis

Descriptive epidemiological methods and spatial statistical analysis were used in this study, and spatial correlation and agglomeration were the key part and our concern. TB registered incidence rate was calculated based on the number of newly registered patients divided by the total population, and *χ*^2^ test was used for rates comparison.

Spatial-temporal analysis was the application of geographic information system technology (GIS), which combined disease data with geographic data to visualize the trend of disease epidemics. Moran’ s index was used to measure the spatial auto-correlation between the meso-regions at an aggregate level [14], and a Poisson model was used to perform space-time scan and to detect any spatial-temporal cluster by SaTScan v9.6 and ArcGIS 10.8 (ESRI Inc. Haidian, Beijing, China) softwares [15].

The exponential smoothing model could utilize disease data of the past to show the changing pattern of disease, which has the value to predict the future epidemic. In this paper, the model was created by monthly TB cases from 2009 to 2018 and verified by data of 2019, which was evaluated through Ljung-Box Q statistic with a statistically significant level (*α* *=* *0.05*) by SPSS 27.0 [16] (IBM Inc. Armonk, NY, USA).

## Results

### Epidemiological characteristics analysis

43 859 cases were collected by the TBIMS from 2009 to 2019. The average annual TB registered incidence in Qinghai was 70.00/100 000, with a downward trend in 2009–2014 and an upward inclination in 2014–2019 (*χ*^2^ *=* *1663.85, P* < *0.001*). The highest number of cases was recorded almost in March and April for each year, while the lowest was seen practically in February (Fig. 1b). It was demonstrated that the average annual registered incidence in Yushu and Golog both exceeded 150.00/100 000 in 2009–2019, and then Huangnan was the area which registered incidence ranked third with the amount of over 100.00/100 000. Besides, the registered incidence of some counties in Yushu and Golog was over 300.00/100 000 in recent 2 years (Fig. 1a). In terms of sex, the registered incidence of TB was higher in males with ratio 1.27: 1 (Male: Female). It was higher in farmers and herdsmen by occupations and Tibetans was higher by ethnicity. And it also reflected in the elderly (the age older than or equal 65) (Table 1) when age was taken into account, however, the largest proportion for each year from 2009 to 2019 was observed in age of 40 (2009), 37 (2010), 40 (2011), 20 (2012), 50 (2013), 18 (2014), 18 (2015), 18 (2016), 17 (2017), 21 (2018), 19 (2019), which suggested young people might have more opportunities to be exposed to tuberculosis infection.

**Table 1.** The annual TB incidence (per 100 000 population) of 2009–2019 with demographic characteristics in Qinghai province

Demographic factors	2009	2010	2011	2012	2013	2014	2015	2016	2017	2018	2019
Sex											
Male	91.68	87.49	80.54	77.29	75.44	52.81	58.31	61.54	77.56	88.03	101.78
Female	76.64	62.78	57.58	55.90	55.45	43.25	49.80	52.47	69.46	72.37	92.41
Occupation											
Farmer and herdsman	275.88	252.15	242.25	235.42	245.40	178.05	206.27	216.80	269.24	284.24	327.94
Student	23.62	22.56	22.56	32.97	24.66	19.32	30.14	46.42	68.59	73.49	85.96
Health care worker	91.20	68.68	62.38	53.58	61.60	30.01	35.00	26.61	29.48	53.22	42.88
Others	30.39	26.09	22.20	21.05	19.40	14.68	11.44	9.83	13.46	19.35	28.19
Ethnicity											
Han nationality	73.08	58.82	52.40	48.67	48.25	32.57	41.61	41.85	48.33	45.96	44.41
Tibetans	142.01	123.60	119.62	121.07	116.49	92.43	93.43	102.38	145.33	168.08	223.87
Hui nationality	60.79	67.98	57.92	55.06	55.43	40.57	41.05	45.72	48.06	56.20	65.34
Others	46.50	45.11	43.14	38.24	45.34	27.57	31.34	29.10	51.22	59.78	79.91
Age											
0–14	5.55	5.48	5.49	7.35	3.09	3.79	3.25	6.02	9.24	14.58	18.34
15–64	94.76	84.54	78.07	73.47	75.16	43.67	58.60	60.21	76.47	85.47	100.98
⩾65	219.23	201.16	179.06	186.36	202.64	246.24	171.19	190.31	253.56	238.59	314.48

### Intractable cases analysis

Quite a number of the patients were more intractable among 43 859 TB cases, in which 1485 cases were recurrent, 415 cases were drug-resistant, 3140 cases were co-infected with other parts of body tuberculosis, 61 cases were co-infected with AIDS, and 2031 cases were co-infected with other diseases apart from AIDS. All case numbers above excluded TB co-infected with AIDS had increased year by year.

### Spatial-temporal characteristics analysis

In the county-level, spatial correlation illustrated with the Moran’ s index for each year from 2009 to 2019 was 0.34 (2009), 0.33 (2010), 0.36 (2011), 0.32 (2012), 0.22 (2013), 0.15 (2014), 0.22 (2015), 0.35 (2016), 0.38 (2017), 0.28 (2018), 0.47 (2019). Space-time scan analysis showed three likely clusters based on annual cases. The most likely cluster was located in central and southern Qinghai in 2009–2010, in which the relative risk (RR) that people infected with TB was 5.72 (*P* *<* *0.001*). The actual number of TB patients was 5397, which far more than the expected number of 1047.29. The other two secondary likely clusters were statistically significant (*RR* *=* *5.04,P* *<* *0.001; RR* *=* *2.14,P* *<* *0.001*) (Table 2).

**Table 2.** Space-time clusters of TB cases with significant higher risk from 2009 to 2019 in Qinghai Province

Cluster	Time frame	Radius (km)	Location	Cases	Expected	RR	*P*-value
Most likely cluster	2009–2010	490.97	Guoluo (Maqin, Banma, Gande, Dari, Jiuzhi, Maduo) Yushu (Yushu, Zaduo, Chengduo, Zhiduo, Nangqian, Qumalai) Haixi (Geermu, Dulan)	5397	1047.29	5.72	<0.001
Secondary likely cluster	2009–2010	171.09	Haibei (Qilian, Haiyan, Gangcha) Haixi (Tianjun)	1042	210.64	5.04	<0.001
2nd secondary likely cluster	2017–2019	92.60	Huangnan (Tongren, Jianzha, Zeku, Henan) Hainan (Tongde, Guinan)	1887	900.10	2.14	<0.001

RR, relative risk; P-value, for Poisson test.

### Exponential smoothing model application

**Fig. 2.**
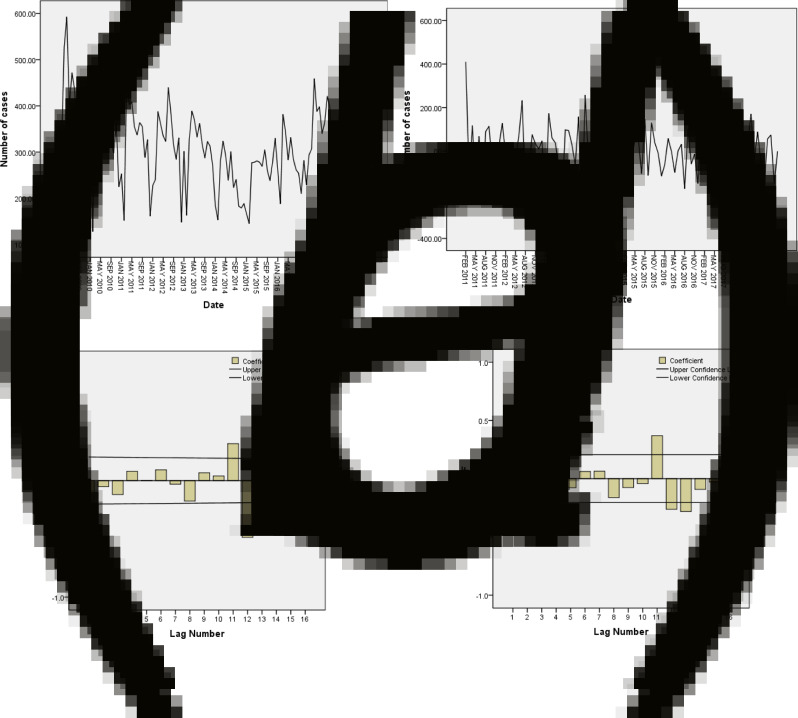
Exponential smoothing model application of Tuberculosis in Qinghai Province. (a) Time sequence of TB cases from 2009 to 2018 in Qinghai Province. (b) Time sequence of TB cases from 2009 to 2018 after transforming and differencing in Qinghai Province. (c) Autocorrelation function (ACF) of the monthly TB cases in Qinghai Province. (d) Partial autocorrelation function (PACF) of the monthly TB cases in Qinghai Province.

The monthly cases vary from month to month with a fluctuation, which conformed to the requirement of the time series analysis after exchanging of differences and transformation including one order difference and two order seasonal differences (Fig. 2). Then three seasonal models were created through the Expert Modeler, and we selected the exponential smoothing Winters' additive model as the best one after comparing the value of the Stationary *R*^2^ Substitute original data for fitting, which was calculated through substituting original data into the model for fitting (Table 3).

**Table 3.** Exponential smoothing model fitting for the TB cases from 2009 to 2018 in Qinghai Province

Model	Stationary *R*^2^	*R*^2^	RMSE	MAPE	MAE	MaxAPE	MaxAE	Normalized BIC	*P*-value (Ljung-Box Q)
Winters' additive model	0.65	0.69	50.49	13.84	92.47	39.33	146.11	7.96	0.79

RMSE, Root mean square error; MAPE, Mean absolute percentage error; MAE, Mean absolute error; MaxAPE, Max absolute percentage error; MaxAE, Max absolute error; BIC, Bayesian Information Criterion.

We applied the exponential smoothing Winters' additive model to predict monthly TB cases in 2019 and that was verified. The predicted values were basically consistent with the observed ones. The monthly TB cases could be predicted in future for TB control.

## Discussion

We summarized epidemiological characteristics of 43 859 TB patients registered from 2009 to 2019 in Qinghai, which is the largest spatial epidemiological study of TB with the longest period in this province.

The study demonstrated the highest number of cases was recorded almost in March and April annually, while the lowest was seen in February. The potential reasons could be: firstly, Qinghai lies in western China where is very cold, therefore local residents prefer staying at home rather than doing activities outdoors, which make people have not much fresh air. Secondly, a dry climate could bring about the delayed increasing trend for the morbidity of TB [17]. Heating begins in October and lasts for months in Qinghai because of the cold, and could exacerbate the dryness of the climate, which probably resulted in more TB patients showed up in next March and April. Thirdly, the incidence of respiratory diseases was higher in Spring, which drove more people suffering from suspicious respiratory diseases and going to a hospital for diagnosis, thus it is helpful for early diagnosis of TB patients with mild symptoms and those in the incubation period and asymptomatic infected individuals. Fourthly, the annual medical checkups of employees in many companies and institutions started in March, which increased the number of diagnosed TB patients. Fifthly, Chinese New Year usually falls in February, which is a holiday season for Chinese people to visit relatives and friends, thus increased the chances of close contact during festival gatherings, those latent or undiagnosed TB patients might spread germs to others. In addition, less doctors and health workers making a shift in hospitals during the festival holiday and less potential patients going to see doctors, which might be a reason why less cases were recorded in February and more cases were confirmed in March or April.

The results suggested that the distributions of TB were spatially correlated throughout entire Qinghai, higher morbidity was seen in the south, especially in prefectures of Yushu and Golog, where TB prevention strategies and measures were not fulfilled well due to scattered population, inconvenient traffic and frequent geological hazards, which probably resulted in the formation of the case clusters. Gerermu had a higher TB incidence in 2009 and managed to a lower one in the last 2 years for case effective management and early detection.

The results showed higher incidences of TB among males, farmers and herdsmen, elderly people and Tibetans. Males usually have more unhealthy lifestyles (smoking, alcohol abuse) which could increase the risk of TB infection. Fu H [18] reported that low immunity and increased life expectancy probably led to a higher TB prevalence in the elderly. Our study also found that young people had a large proportion of TB cases, which we speculated that young people might have more chance exposed to tuberculosis infection. Take students as an example, the higher TB registered incidence was shown among students from 2014 to 2019 (Table 1). Qinghai has vast pastoral areas with a sparse population, so most schools are boarding schools. Students live and study together day and night, which increases the opportunities of tuberculosis infection [19]. In 2012, an outbreak of TB in two schools of Qinghai province came from the same source， who was a recurrent TB patient [20]. We also noticed some TB cases came from health care workers, but fortunately, it decreased year by year, which may probably relate to their increased self-protection awareness, intensive protective measures and improved facilities, that reduced occupational and iatrogenic infections.

In this study, the highest TB proportions were farmers and herdsmen and Tibetans. All six Tibetan autonomous prefectures in this province are also the main agricultural and pastoral areas, so most Tibetans living in these areas were farmers or herdsmen occupationally, who raise cattle and sheep for living and usually eat more beef and mutton. Olea-Popelka [21] reported that the control of animal TB could affect the epidemic of TB in human beings. Farmers and herdsmen had more possibility of being exposed to diseased animals due to their diet and daily contact with livestock, which probably increased their TB infection.

Over 10% of patients were intractable, included recurrent, drug resistant, or co-infected with other diseases. It was reported that treatment cost for drug-resistant TB patients was several times higher than that of ordinary patients [22, 23]. The TB co-infected with other diseases led to the treatment tougher and costly, especially with AIDS, could cause higher mortality [24]. All these cases undoubtedly aggravated the local tuberculosis situation and economic burden. Governments and CDC at all levels should increase investment in tuberculosis prevention and control, strengthening surveillance, finding early patients, preventing drug resistance and co-infection.

The exponential smoothing model has the advantage of forecasting infectious diseases, which could be applied for predicting the monthly and annual TB cases thus to improve TB prevention and control in this province in future.

## Conclusion

This research concluded that the epidemic of TB in Qinghai is still challenging and it has a long way to go for controlling TB. Our findings may shed light on developing better strategies for TB control in this region and determining the priority of tuberculosis prevention and treatment.

## Data Availability

The demographic data that support the findings of this study are openly available in Qinghai Provincial Statistical Yearbook at http://tjj.qinghai.gov.cn/tjData/qhtjnj/ [13]. The registered tuberculosis cases data that support the findings of this study are available from Qinghai Center for Disease Prevention and Control. Restrictions apply to the availability of these data, which were used under license for this study. Data are available from Binzhong Ma with the permission of Qinghai CDC.
